# The effects of nitrous oxide on vascular endothelial growth factor (VEGF) and its soluble receptor 1 (VEGFR1) in patient undergoing urological surgery

**DOI:** 10.12669/pjms.301.3197

**Published:** 2014

**Authors:** Yasemin Hakimoglu, Murat Can, Sedat Hakimoglu, Ayca Gorkem Mungan, Sereften Acikgoz, Nuran Cikcikoglu Yildirim, Necmettin Aydin Mungan, Isil Ozkocak Turan

**Affiliations:** 1 Yasemin Hakimoglu, MD, Department of Biochemistry, Hatay Antakya State Hospital, Hatay, Turkey.; 2 Murat Can, MD, Associate Professor, Department of Biochemistry, Bulent Ecevit University, Medical Faculty, Zonguldak, Turkey.; 3 Sedat Hakimoglu, MD, Assistant Professor, Department of Anesthesiology and Reanimation, Mustafa Kemal University, Medical Faculty, Hatay, Turkey.; 4 Ayca Gorkem Mungan, MD, Associate Professor, Department of Biochemistry, Bulent Ecevit University, Medical Faculty, Zonguldak, Turkey.; 5 Sereften Acikgoz, MD, Associate Professor, Department of Biochemistry, Bulent Ecevit University, Medical Faculty, Zonguldak, Turkey.; 6 Nuran Cikcikoglu Yildirim, PhD, Department of Environmental Engineering, Tunceli University, Faculty of Engineering, Tunceli, Turkey.; 7 Necmettin Aydin Mungan, MD, Professor, Department of Urology, Bulent Ecevit University, Medical Faculty, Zonguldak, Turkey.; 8 Isil Ozkocak Turan, MD, Professor, Department of Anesthesiology and Reanimation, Bulent Ecevit University, Medical Faculty, Zonguldak, Turkey.

**Keywords:** Nitrous oxide, VEGF, VEGFR1, Urological Surgery

## Abstract

***Objective:*** Anesthesia and surgical intervention, leads to the development of systemic inflammatory response. The severity of the inflammatory response depends on the pharmacological effects of anesthetic agents and duration of anesthesia. Objective of the study was to investigate the effect of nitrous oxide on VEGF and VEGFR1 levels in patients undergoing surgery.

***Methods:*** Forty-four patients undergoing elective urological surgery were included in the study. Anesthesia maintenance was provided with 1-2 MAC sevoflurane, O_2_ 50%, N_2_O 50% in 4L/m transporter gase for group 1 (n=22) and 1-2 MAC sevoflurane, O_2_ 50%, air 50% in 4L/m transporter gase for group 2 (n=22) Venous blood samples for the measurement of VEGF and VEGFR1 were taken before the induction of anaesthesia, 60 minutes of anesthesia induction, at the end of anaesthesia and 24 hours after operation. In statistical analysis Bonferroni test and analysis of variance at the repeated measures were used

***Results:*** In the postoperative period serum VEGF levels had decreased significantly in both group whereas VEGFR1 did not show a significant change.

***Conclusions:*** Nitrous oxide showed significant effect on angiogenic parameters. Further detailed studies are required to evaluate the effect of nitrous oxide.

## INTRODUCTION

The vascular system is essential for providing oxygen and nutrients, removing metabolic waste products, and furnishing efficient access of leukocytes to tissues throughout larger animals. Angiogenesis, the sprouting of new capillaries, is required for the development of the vascular system and, consequently, the growth of vertebrates.^1^

Vascular endothelial growth factor (VEGF) is a pivotal regulator of angiogenesis and is suggested to play an important role in tumor growth and dissemination.^2^^,^^3^ Besides being a tumor growth-promoting factor, VEGF is also characterized as an inflammatory molecule.^4^ It is synthesized by a variety of inflammatory cells including endothelial cells, megakaryocytes^5^, granulocytes,^6^ activated lymphocytes,^7^ and macrophages^8^ and may be released from these cells following activation.^9^ Thereby VEGF may contribute to local hyperpermeability of traumatized or inflamed tissue as well as induce revascularization of the inflamed area.

Several receptors for VEGF have been identified.^10^ Of these the soluble form of VEGFR1 (sVEGFR1) is presumed to antagonize the effects of VEGF by a direct competitive binding of VEGF.^11^

Nitrous oxide (N_2_O; laughing gas) has been widely used in clinical practice for decades because its effective analgesic properties are achieved at concentrations below those required for general anesthesia.^12^ Nevertheless, several adverse effects, including megaloblastic anemia, homocysteinemia and its possible risk for atherosclerosis, thrombosis, cognitive dysfunction, neurotoxicity, possible teratogenicity, increased intracranial pressure and cerebral blood flow, expansion of air spaces and hypoxia, post-operative nausea and vomiting, and possible immunosuppression are known.^13^

Surgical trauma is followed by inflammatory and wound healing processes, with local release of various growth factors including VEGF.^14^^,^^15^ In animal models, surgery, trauma and inflammation have been shown to facilitate angiogenesis, tumour growth and metastasis, the effect being both local and systemic.^16^^,^^17^

The stress response to surgery is a major neuroendocrine and cytokine response to surgical trauma, characterized by increases in catecholamine and steroid hormones, with predictable metabolic consequences, including hyperglycemia and negative nitrogen balance.^18^ It is possible that cytokines increased by the surgical stress response may be linked with some of the angiogenic factors associated with elective urological surgery.

The aim of the present study was to investigate the influence of nitrous oxide on VEGF and VEGFR1 in order to detect any effect that this agent might have on an inflammatory response.

## METHODS

This study was approved by Ethical Committee at Karaelmas University Application and Research Hospital CM, dated January 15^th^, 2009. Forty four Society of Anesthesia (ASA) risk scores I-III adult patients who were between 18 and 65 years and were to undergo an elective urological surgical initiative that would last 1 to 4 hours in the main operation room at Karaelmas University Application and Research Hospital were included. The study was conducted between January 2009 and January 2010. Patients with chronic metabolic diseases, liver failures and acute anemia were not included.

All the patients were applied the standard IM midazolam (0.07 mg/kg) premedication one hour before the surgery. Blood samples were taken from the patients prior to anesthesia in order to determine their VEGF, VEGFRl levels. Before induction, all the patients were applied 5-7 ml/kg Ringer Laktat fluid replacement. All patients were preoxygenated with 10 L/dk 100% of oxygen for one minute. Anesthetic induction was done with 2.4 mg/kg of propofol and 1 µg/kg of fentanyl. Intubation was done three minutes after 0.6 mg/kg of rocuronium was given as a muscle relaxant agent. Following the intubation, a high current of 6 L/dk was applied for 5 minutes.

Maintenance of anesthesia was done with 1-2 MAC sevoflourane in group 1 (n=22), keeping O_2_ at 50% and N_2_O at 50% and with 1-2 MAC sevoflourane in group 2 (n=22) under 4 L/dk carrier gas, keeping O_2_ at 50% and air at 50%. Maintenance analgesia need was met in group 2 with 1 mcg/kg/saat fentanyl infusion. Ventilation tidal volume (TV) was kept at 6-8 ml/kg, I:E ratio at 1:2 and respiratory frequency was maintained in a way to keep ETCO_2_ at 35-40 mmHg, which makes normocapnia possible. FiO_2_ value was preserved between 30 and 35%. Anesthetics were stopped and patient was ventilated with 100% of O_2_ when the last skin suture was begun before the operation ended. Muscle relaxant was antagonised with 0.05 mg /kg of neostigmin and 0.01 mg/kg of atropine. The patient was respirated by hand after the antagonists were applied and spontaneous respiration was controlled every ten seconds In order to determine the levels of VEGF and VEGFR1, blood samples were taken. from patients

After vascular access was obtained, blood samples were taken from all the patients before anesthesia (Pre-operative 0^th^ min), at the 60^th^ min of anesthesia (Intra-operative 60^th^ min), after anesthetic agents were stopped (Post-operative 0^th^ min) and at Post-operative 24^th^ hr. After the blood samples were congealed, they were centrifuged at 3500 rpm for five minutes. The serums separated after centrifuges were kept to be analysed at - 80C.

***VEGF measurement: ***VEGF levels were assayed by an enzyme-linked immunosorbent assay (ELISA) kit (Catalog No: KHG0112: 1 plate, Camarillo, USA). Detection limit of the assay is <5 pg/ml.

***VEGFR1 measurement: ***VEGFR1 levels were assayed by an enzyme-linked immunosorbest assay (ELISA) kit (Catalog No: BMS268/2: 1 plate, Vienna, Austria). Detection limit of the assay is 6 ng/ml.

***Statistical analysis: ***Statistical evaluation was done using the SPSS 18.0 program. Descriptive statistics were expressed as mean ± standard deviation for numeric data and as number and percentage for categorical data. Compatibility of the measured variables with normal distribution was examined with the Kolmogorov-Smirnov test. Significance test of the difference between two means was used for the differences of measured variables between the groups. Differences of categorical variables between the groups were evaluated with chi-square test. Changes in measured variables in time for the repetitive measurements were examined with single direction variance analysis.

<

## RESULTS

The measurement values of VEGF and VEGFR1 belonging to the group of nitrous oxide-administered patients at preop, intraop 60th minute, postop 0th minute and postop 24th hours are given in Table-I.

**Table-I:** The significance of change in parameters depending on time in the nitrous oxide-administered patient group^*^

	*Preop.* *(0th minute)*	*Intraop.* *(60th minute)*	*Postop.* *(0th minute)*	*Postop.* *(24th hours)*	*P*
VEGF (pg/ml)	364.06153.66	262.88136.91	237.72136.91	233.64120.01	**<0.001**
	(87.7-750.0)	(57.7-750.0)	(63.6-625.3)	(38.7-518.1)	
soluble VEGFR1(ng/ml)	0.180.06	0.220.05	0.190.06	0.270.26	0.159
(0.06-0.338)	(0.112-0.298)	(0.084-0.379)	(0.003-1.383)

^* ^The results were indicated as MeanSD N=22

 The change in VEGF depending on time in the nitrous oxide-administered patient group was found to be significant (p<0.001). VEGF levels in preop. 0^th^ min is statistically higher than other times. There was no significant difference between the levels of VEGF at other times.

No significant difference between times in terms of VEGFR1 was found in the group of patients who receive nitrous oxide (p=0.159). The measurement values of VEGF and VEGFR1 belonging to the group of patients who were not administered nitrous oxide at preop, intraop 60th minute, postop 0.min and postop 24th hours are given in Table-II.

**Table-II:** Significance of the change in parameters in the group of patients who were not administered nitrous oxide depending on time^*^

	*Preop. * *(0th minute)*	*Intraop. * *(60th minute)*	*Postop. * *(0th minute)*	*Postop. * *(24th hours)*	*P*
VEGF (pg/ml)	95.9545.97	86.3047.10	82.4447.22	82.7535.21	**0.013**
	(40.5-209.7)	(25.7-192.7)	(22.2-207.6)	(33.4-159.4)	
Soluble VEGFR1(ng/ml)	0.22018	0.200.08	0.220.09	0.210.009	0.334
(0.1-1.0)	(0.1-0.4)	(0.1-0.4)	(0.1-0.5)

^* ^The results were indicated as Mean SD N=22

 The change in VEGF depending on time in the group of patients who did not receive nitrous oxide was found to be significant (p=0.013). The difference between the levels of VEGF at Preop 0^th^ minute and those at all the other times was found to be significant. There was no significant difference between the levels of VEGF at other times.

 No significant difference between times in terms of VEGFR1 was found in the group of patients who did not receive nitrous oxide (p=0.334). 

## DISCUSSION

Angiogenesis, or the formation of new blood vessels from pre-existing ones^19^ plays a pivotal role during embrional development and later, in adult life, in a variety of physiological and pathological conditions, such as malignancy and chronic inflammation.^20^ Diseased or injured cells in response to genetic alterations, hypoxia, hypoglycemia, mechanical stress, and/or inflammatory proteins, release pro-angiogenic growth factors such as vascular endothelial growth factor (VEGF) into the surrounding tissue.^21^ VEGF is the major endogenous regulator of endothelial cell proliferation, migration, and differentiation.^22^ Angiogenesis and VEGF are indistinguishable.^23^

Svendsen et al (2005) investigated that the effect of major and minor surgery on variations in sVEGF and sVEGFR1 concentrations in vivo and on bacterial antigen induced release of sVEGF and sVEGFR1 from whole blood in vitro. They suggested that plasma sVEGF and sVEGFR1 concentrations did not change during surgery and in vitro bacterial stimulation led to increased release of sVEGF, which was not compensated for by an equivalent increase in sVEGFR1.^24^

Svendsen et al (2005) also evaluated perioperative plasma concentrations of soluble VEGF (sVEGF) and soluble VEGFR1 (sVEGFR1) in patients undergoing elective colectomy. They found that the major surgical trauma led to significant intra- and postoperative changes in sVEGF and sVEGFR1. The high preoperative levels were significantly decreased 30 days after surgery. The intra- and postoperative changes of both molecule levels were similar in patients undergoing laparoscopically assisted and open colectomy.^25^

An insignificant decrease was observed on the post-operative day one in the study in which Futami R et al investigated serum VEGF levels following a major surgical damage. However, there was an increase in the serum VEGF levels after the day one. It was reported that this increase peaked on the postop day 14. Researchers suggested that the postoperative VEGF increase might be the angiogenetic response to tissue repair.^26^ Compared with preop levels, VEGF levels were found to be low at postop 4 and 24 hours in the 30 patients who had mastectomy operation under general anasthesia using nitrous oxide but no statistically significant difference was found.^18^ A significant decrease was seen in VEGF levels in patients with oesophagus cancer on day one following surgical trauma. VEGF levels started to increase on day three and receded to preop levels on day five.^27^

VEGF and soluble VEGFR1 levels were examined in a study in which 61 patients of abdominal surgery who were given general anaesthesia without giving them nitrous oxide. A decrease was detected in Postop VEGF and soluble VEGFR1concentrations but this decrease was not statistically significant.^24^

In another study which investigated the effect of open and laparoscopic colectomy on VEGF and soluble VEGFR1, a significant fluctuation in VEGF and soluble VEGFR1 levels was detected depending on time. While serum VEGF levels were at their lowest at intraop hour 1, there was an increase after the operation and VEGF levels receded to preop concentrations on the postop day 30. When serum soluble VEGFR1 levels were compared with preop levels, a decrease was seen at the intraop hours 1, 2 and 6. VEGFR1 levels dropped below preop levels on the day 30.^25^

In our study, VEGF levels decreased in both groups during the surgery. However, the fact that İntra-operative 60^th^ min. according to Pre-operative 0^th^ min VEGF levels decreased (28%) more in the group who were given nitrous oxide than the group (11%) who were not given nitrous oxide suggests that the decrease occurred due to nitrous oxide. The fact that there was not a significant change in serum soluble VEGFR1 levels in both groups shows that soluble VEGFR1 did not contribute to the decrease in VEGF levels. These results suggest that the decrease in VEGF levels were due to dilutional effect arising from stress response.

## Authors Contribution:

YH conceived, designed and did statistical analysis and editing of manuscript. MC was involved in clinical management of patients. SH did data collection and manuscript writing. NCY, SA & AGM contributed in manuscript writing. NAM & IOT did review and final approval of manuscript.
